# Infections sexuellement transmissibles tropicales Synthèse de la Journée scientifique de la SFMTSI du 9 novembre 2023

**DOI:** 10.48327/mtsi.v3i4.2023.447

**Published:** 2023-10-24

**Authors:** Éric CAUMES

**Affiliations:** Sorbonne Université, Paris

**Keywords:** Infections sexuellement transmissibles, IST, Monkeypox, Zika, Ebola, HPV, Chancre mou, Donovanose, LGV, Syphilis, Résistance aux antibiotiques, *Neisseria gonorrhoeae*, *Mycoplasma genitalium*, *Treponema pallidum*, Prophylaxie pré-exposition, PrEP, Migrants, Sexually transmitted infections, STI, Monkeypox, Zika, Ebola, HPV, Soft chancre, Granuloma inguinale, LGV, Syphilis, Antibiotic resistance, *Neisseria gonorrhoeae*, *Mycoplasma genitalium*, *Treponema pallidum*, Pre-exposure prophylaxis, PrEP, Migrants

## Abstract

S'intéresser aujourd'hui aux infections sexuellement transmissibles (IST) tropicales, c'est appréhender les risques de demain. L'arrivée de la variole du singe, à l’été 2022, a confirmé le risque d'exportation, par les voyageurs, d'IST aussi tropicales que l'infection VIH/sida a pu l’être à son origine. Certains dangers sont déjà bien identifiés. Les IST classiques restent nombreuses sous les tropiques et rien n'indique qu'elles sont en décroissance. La résistance aux antibiotiques est croissante pour le gonocoque, déjà bien installée pour *Mycoplasma genitalium,* et en devenir (incertain) pour la syphilis. Certaines viroses tropicales (Zika, Ebola, variole du singe) peuvent aussi être sexuellement transmissibles, et parfois des mois après la guérison (Ebola). Dans ce contexte, l’élargissement des indications de la PrEP aux migrants, et sa diffusion en Afrique méritent d’être discutés au-delà des cercles traditionnels.

## Abstract (en)

Sexually transmitted infections (STIs) deserve more attention today than ever in the tropics. Indeed, the emergence of monkeypox in 2022 in Western countries reminds the risk of exporting STIs from the tropics as it was already known for decades with the worldwide spread of HIV/AIDS infection from tropical Africa. Some hazards are already well identified. According to WHO 2023 report STIs are increasing in the world. Antibiotic resistance is increasing for *Neisseria gonorrhoeae,* and already well established for *Mycoplasma genitalium,* whereas *Treponema pallidum* has become resistant to macrolides within the last twenty years. Some neglected tropical diseases (Zika, Ebola, monkeypox) can also be sexually transmitted, sometimes months after cure (Ebola). In this setting, the use of PrEP in migrants, and in Africa, is worth to be discussed beyond traditional circles.

La journée a été découpée en 4 parties, avec au total 12 interventions censées couvrir tous les aspects importants des infections sexuellement transmissibles (IST) en milieu tropical. La première partie de la journée a été consacrée aux **IST tropicales émergentes**.

Un bilan de l’épidémie d'infection à **Monkeypox** dans les pays occidentaux a été dressé par le **Dr Pascal Del Guidice** (Fréjus-St Raphaël). Il a été rappelé comment le virus, autrefois cantonné à un réservoir animal, a d'abord émergé en Afrique centrale, puis en Afrique de l'Ouest, avec un important foyer épidémique au Nigeria à partir duquel plusieurs cas sporadiques ont été exportés hors d'Afrique (Singapour, Israël, Royaume-Uni) au tournant des années 2020. L’épidémie de 2022, toujours inexpliquée à ce jour, a d'abord touché l'Europe et l'Amérique, à peu près en même temps, avant de diffuser au reste du monde dans les semaines suivantes. Elle a presque exclusivement touché les hommes, notamment les hommes ayant des relations sexuelles avec des hommes (HSH). En 2023, l’épidémie a reflué et on observe maintenant des cas sporadiques. Du bilan de l’épidémie, on retient l'importance variable du portage asymptomatique (5-50% selon les séries), la faible létalité (< 0,01%), l'existence de formes graves chez les immunodéprimés (notamment les patients infectés par le VIH avec des CD4 bas et une charge virale élevée), des atteintes viscérales rares mais potentiellement sévères (myocardites), des formes chroniques (cutanées, ophtalmologiques), des formes récurrentes chez des personnes déjà infectées et des échecs de la vaccination (avec des formes mineures). Le virus est bien présent dans les lésions cutanéo-muqueuses d'où il peut être excrété pendant plusieurs jours, notamment dans le sperme. Le temps de clairance du virus du sperme a été estimé à 13 jours chez 50% des patients et 53 jours chez 95% d'entre eux. L'efficacité vaccinale anti-variole a été estimée à 86%. Le retour au « safer sex » chez 50% des HSH et la campagne de vaccination expliquent la décroissance de l’épidémie.

Le **Dr Paul-Henri Consigny** (Centre médical de l'Institut Pasteur, Paris) est revenu sur l’épidémie de **Zika**. Il a rappelé les grandes étapes de la progression de l’épidémie, de l’île de Yap (Micronésie, 2007), à la Polynésie Française (2013), à l’île de Pâques (2014), à l'Amérique du Sud (2015) puis aux Antilles (2016). La maladie est toujours endémique dans nombre de pays dont le Brésil, mais le niveau de circulation virale est faible et les cas importés sont devenus rares. Du bilan de l’épidémie, on retient l'importance des formes asymptomatiques (50-80% des cas), la rareté des formes neurologiques (< 0,1%), la faible létalité (< 0,01%) et le risque de malformations congénitales (plus élevé au 1^er^ trimestre de la grossesse) revu à la baisse. Estimé à 7% lors de l’épidémie en Guadeloupe, ce risque est maintenant évalué à 1,6%. La transmission sexuelle est connue depuis 2008 (article publié en 2013), le virus a été identifié dans le sperme et les cas se sont multipliés chez les partenaires sexuels de voyageurs, au retour de zones d'endémie, en 2015-1016. L'infection se transmet principalement des hommes vers les femmes, mais aussi des femmes vers les hommes et d'hommes à hommes. Le virus est retrouvé dans le sperme par PCR chez environ 1/3 des malades et la PCR peut rester positive chez 21% des patients jusqu’à 90 jours mais des durées de clairance virale plus prolongées ont été observées (le record est de 281 jours). Les principaux facteurs de risque d'excrétion virale prolongée sont un âge élevé et un nombre faible d’éjaculations. Néanmoins, il faut distinguer la PCR de la culture virale qui semble se négativer avant 30 jours même si un cas a été décrit jusqu’à 69 jours. Chez la femme, le virus est retrouvé dans les sécrétions vaginales dans moins de 5% des cas. Cette potentielle transmission sexuelle et le risque de transmission maternofœtale expliquent l'existence de recommandations qui concernent les femmes enceintes, les femmes avec désir de grossesse ou en âge de procréer, et les hommes ayant une femme avec désir de grossesse. Elles ne sont plus beaucoup respectées, même dans les pays d'endémie.

Le **Pr Denis Malvy** (Bordeaux) a rappelé que le virus **Ébola** était transmis sexuellement, de façon prolongée après la guérison, étant ainsi à l'origine de résurgences épidémiques à partir de convalescents. Il a précisé les modalités d'excrétion virale du virus Ébola dans le sperme, évaluées par PCR: 73% des patients sont positifs au 1^er^ prélèvement (médiane de 55 jours après le début des signes), 50% sont encore détectables à J108, et 10% à J325, le record étant établi à 408 jours. Au cours de la résurgence de l’épidémie en Guinée-Conakry, le patient à l'origine de la chaîne de transmission était considéré comme guéri depuis 482 jours lors de la contamination de sa partenaire sexuelle, et il a excrété dans le sperme un virus viable jusqu’à 531 jours. Il s'agit donc bien de virus infectant et qui peut être transmis longtemps après la guérison clinique. Des conseils de « safer sex » sont donc donnés aux convalescents et l'OMS recommande un suivi par PCR de l'excrétion virale dans le sperme jusqu’à négativation de deux PCR consécutives à un mois d'intervalle. L'orateur a néanmoins souligné que ces recommandations étaient difficiles à appliquer étant donné la stigmatisation dont ces malades font l'objet.

La deuxième partie de la journée a été consacrée à des **IST diverses,** plus emblématiques des tropiques (chancre mou, donovanose, lymphogranulome vénérien), ou des IST historiques (syphilis, HPV).

Le **Pr Antoine Bertolotti** (La Réunion) a couvert l'infection par les **Papillomavirus humains** (HPV), en soulignant d'emblée que l'on n'en savait pas grand-chose en milieu tropical en dehors de la complication terminale qu'est le cancer du col de l'utérus à l'origine de 625 000 cas et de 340 000 morts en 2020 dans le monde. Il a rappelé qu'il existait aujourd'hui plus de 450 sous-types de virus HPV et qu'en dépit du grand nombre de condylomes (la forme clinique la plus fréquente de l'infection), les recommandations thérapeutiques restaient encore discutées et non consensuelles (des recommandations sont annoncées pour 2024 en France). Il a fait un tour d'horizon des différentes formes d'infection par les HPV (verrues, condylomes, cancers des muqueuses génitales, anales et orales), en insistant sur les prédispositions génétiques et virologiques (type d'HPV) aux formes graves d'infection par HPV (maladie de Heck, papillomatose orale floride, tumeur de Buschke Lowenstein, papulose bowenoïde, épidermodysplasie verruciforme, verrues profuses et « homme arbre »). Il a conclu en rappelant la faiblesse de la couverture vaccinale dans les pays tropicaux et en France (38% chez les jeunes femmes) comparativement à d'autres pays (Australie…) où les cancers HPV induits sont en voie de disparition.

Le **Pr Jean-Jacques Morand** (Toulon) a fait le point sur trois IST tropicales emblématiques. Le **chancre mou** semble ne plus être un problème de santé publique. Par contre, l'agent pathogène (*Haemophilus ducreyi*) est une cause probable d'ulcération cutanée chez les enfants dans des zones de tréponématoses endémiques avec lesquelles il partage certaines caractéristiques cliniques dermatologiques. La **donovanose** semble également ne plus être un problème de santé publique; elle apparaît même éradiquée d'Afrique du Sud. En revanche, moins de 10 cas par an ont été observés chez les militaires américains entre 2011 et 2020. La **lymphogranulomatose vénérienne** est une IST ré-émergente chez les HSH. Le portage pharyngé ou anal asymptomatique ou peu symptomatique apparaît fréquent, estimé de 15% (dépistage par PCR) à 70% (dépistage par sérologie). En dehors de cette population, seuls des cas sporadiques sont décrits dans les pays autrefois d'endémie.

Le **Pr Pierre Couppié** (Cayenne) a présenté les particularités de la **syphilis** dans les pays tropicaux. L’épidémiologie est différente de celle observée dans les pays occidentaux avec une prédominance d'hommes mais beaucoup moins marquée qu'en Occident, une transmission hétérosexuelle prédominante, et le risque de syphilis congénitale du fait du plus grand nombre de cas chez les femmes. Elle représente 10% des cas mondiaux de syphilis, avec une estimation de 700 000 cas/an selon l'OMS. Le dépistage systématique des femmes enceintes reste la meilleure prévention de la syphilis congénitale. L'intervenant a illustré par des photographies les particularités sémiologiques de la syphilis sur peau noire. Il a rappelé que la sérologie dite de syphilis est une sérologie de tréponématose qui, en pays d'endémie de tréponématose non vénérienne, ne permet pas de faire la distinction entre syphilis et tréponématose non vénérienne (pian, bejel ou syphilis endémique et pinta).

La troisième partie de la journée était consacrée à **l’épidémiologie mondiale de la résistance aux antibiotiques.**

La **résistance du gonocoque** a été détaillée par la **Pr Béatrice Berçot** [Centre national de référence (CNR) des IST bactériennes, Paris]. Elle a rappelé les données de l'OMS pour l'année 2020: 374 millions de nouveaux cas d'IST curables (adultes de 19 à 45 ans) avec en tête de liste: trichomonose vaginale, chlamydiose (128,5 millions de cas), gonorrhée (82,4 millions) et syphilis (7,1 millions). Elle a montré que la gonococcie était en constante progression depuis plus de 10 ans en Europe (x 5) et en France (x 10) avec une prédominance des HSH (54% en Europe, 74% en France). L’évolution historique de la résistance aux antibiotiques recommandés pour le traitement des infections à gonocoque depuis 1930 se fait vers l'acquisition très rapide de résistances successives (pénicillines, aminosides, cyclines, azithromycine, céphalosporines orales puis parentérales, fluoroquinolones et maintenant multirésistances) avec le spectre d'une gonococcie totalement résistante aux antibiotiques. Le plus inquiétant est la diminution de la sensibilité (DS) voire la résistance (R) à la ceftriaxone, antibiotique de première ligne, avec une prévalence estimée en 2018 entre 0% et 21%. La Chine et le Sud-Est asiatique sont l’épicentre de cette résistance avec des chiffres élevés: 6% au Vietnam, 1,6% au Laos, 2,4% en Malaisie, 4% aux Philippines, 38% au Cambodge (2020-2021), 2% en Thaïlande (2020-2021), 19% au Japon et 11% en Chine. La résistance varie de 0% à 60% pour l'azithromycine et de 0% à 100% pour les fluoroquinolones. En Europe, en 2022, 3 souches de gonocoques résistantes à la ceftriaxone et à l'azithromycine ont été signalées (Autriche, Royaume-Uni et France) chez des hommes hétérosexuels infectés pour deux d'entre eux au Cambodge. Les espoirs de contrôle de cette infection reposent sur l'arrivée de nouveaux antibiotiques, la vaccination méningocoque B (en attendant un vaccin ciblant le gonocoque), l’épargne des antibiotiques (traiter une infection avec bactérie viable) et la nécessité de test moléculaires de résistance.

**La Pr Cécile Bébear** (CNR des IST bactériennes, Bordeaux) a fait le point sur la **résistance de** ***Mycoplasma genitalium.*** Tous les antibiotiques efficaces (azithromycine et pristinamycine, moxifloxacine, doxycycline et minocycline) font l'objet de résistance à des degrés variables. La résistance aux macrolides (azithromycine) varie de 42% (Pacifique Ouest) à 57% (Europe du Nord). En France, elle est estimée à 51,5% chez les hommes *vs* 24,9% chez les femmes (p < 0,001), 70,8% chez les HSH *vs* 33,2% chez les HSF (p < 0,001), avec une tendance à l'augmentation chez les femmes (p = 0,053). La résistance aux fluoroquinolones varie de 4% (Europe du Nord) à 38% (Pacifique Ouest). En France, la résistance aux fluoroquinolones est stable à 17,1%, estimée à 25,8% chez les hommes *vs* 10,8% chez les femmes (p < 0,001), 39,3% chez les HSH *vs* 22,5% chez les HSF (p > 0,05). Et la résistance combinée (fluoroquinolones, macrolides) atteint 15,9% en 2022. Quant aux cyclines (doxycycline, minocycline), en dépit d'une efficacité in vitro, le taux d’échec est de 60-70% avec la doxycycline, et de 30% avec la minocycline. Ces résistances rendent la démarche thérapeutique particulièrement difficile et il est recommandé en France jusqu’à maintenant d'utiliser les cyclines en 1^re^ intention, puis d'adapter le traitement en fonction des résultats des tests de résistance et de la réponse au traitement.

Le **Pr Nicolas Dupin** (CNR des IST bactériennes, Paris) a fait le point sur la **résistance de** ***Treponema pallidum.*** En préambule, il a rappelé que *Treponema pallidum pallidum* a un petit génome, peu équipé, sans plasmide ni transposon ou bactériophages associés à des transferts de gènes entre bactéries, ce qui limite les risques à la résistance chromosomique. Celle-ci est bien démontrée pour les macrolides du fait de mutations sur le gène de l'ARN ribosomal 23S. La mutation A2058G confère une résistance aux macrolides avec 14 atomes (érythromycine, roxithromycine) et 15 atomes (azithromycine) mais pas aux macrolides ayant 16 atomes. La mutation A2059G confère en plus une résistance aux macrolides ayant 16 atomes (spiramycine, midécamycine et tylosine). Les premières résistances de *T. p. pallidum *aux macrolides sont apparues en 1987. En 30 ans, la résistance est devenue telle (85% de résistance en France) que ces antibiotiques ne peuvent plus être utilisés. L'intervenant a montré comment l'introduction en 2015 de l'azithromycine dans l'algorithme de prise en charge des urétrites avec écoulement en Afrique du Sud s'est accompagnée de l’émergence de souches *T. p. pallidum* ayant une mutation A2058G associée à la résistance aux macrolides. Il a conclu en soulignant le risque de voir se développer des résistances en cas d'utilisation croissante de la doxycycline (prophylaxie post-exposition PEP…) et de l'importance de valider d'autres antibiotiques (nouvelles céphalosporines, oxazolidinones, dalbavancine).

La dernière partie de la journée était consacrée à la **prophylaxie pré-exposition de l'infection VIH (PrEP)** par les antirétroviraux.

Le **Dr Hugues Cordel** (Société française de lutte contre le SIDA, Bobigny) a parlé de la **PrEP chez les migrants**. Il a rappelé les indications classiques de la PrEP chez les HSH, mais aussi chez des « sujets en situation de vulnérabilité exposant à des rapports sexuels avec des personnes appartenant à un groupe à prévalence VIH élevée » (dont font partie certains migrants notamment d'Afrique). L’étude Parcours a montré que les migrants étaient présumés infectés par le VIH (date de contamination inconnue mais extrapolée sur la courbe de décroissance des CD4) avant l'arrivée en France (43% pour les hommes, 59% pour les femmes), autant que dans les 6 premières années qui suivent leur arrivée (31% et 25%, respectivement) et au-delà de 6 ans après l'arrivée en France (26% et 16%, respectivement). Les migrants chez lesquels la PrEP lui paraît plus particulièrement indiquée sont les HSH nés à l’étranger, les personnes transgenres, les migrants qui voyagent dans leur pays d'origine, certaines femmes plus à risque (en situation de vulnérabilité, souvent primo-arrivantes et travailleuses du sexe) ainsi que les migrantes et migrants multipartenaires. Il a discuté les freins au développement de la PrEP chez les migrants ainsi que les leviers qu'il faudrait actionner pour la rendre plus accessible. Il a conclu en faisant le tour des nouveaux médicaments: cabotégravir (injectable à durée d'action prolongée, tous les 2 mois), dapivirine (en anneau siliconé intravaginal), lénacapavir (injectable à longue durée d'action, tous les 6 mois).

**Joseph Larmarange** (Institut de recherche pour le développement, Paris) a discuté la **PrEP en Afrique**. Il a parlé des études faites en Afrique qui ont montré une bonne acceptabilité globale de la PrEP mais d'importants problèmes d'observance chez les plus marginalisés socialement et économiquement, chez ceux ayant choisi la PrEP à la demande (vs continue) et sur le long terme (seuls 2/3 sont encore suivis à M12). Les obstacles identifiés sont la mobilité, la stigmatisation, la peur des effets secondaires, les difficultés liées à une prise quotidienne, l'incompréhension et les idées fausses. Ces difficultés sont bien illustrées par les résultats de l’étude Princess en Côte d'Ivoire: 96% des femmes étaient intéressées par la PrEP à l'entrée de l’étude, 57% sont sorties de soins avant le commencement, 64% de celles qui ont débuté le traitement n'ont pas fait leur première visite de suivi et, parmi celles qui ont fait leur première visite de suivi, 27% ont rapporté avoir arrêté la PrEP depuis la visite d'initiation. Pour lui, la cause de ces problèmes d'observance est la lourdeur du suivi préventif qu'il estime plus importante que celle du suivi curatif. La solution lui semble venir des antirétroviraux à longue durée d'action injectables ou en anneau vaginal. Il conclut en disant qu’« aucun outil ne constitue de solution miracle » et qu'une « innovation efficace sera inefficiente si elle n'est pas adaptée aux contraintes sociales, culturelles, légales auxquelles les populations font face et aux contraintes structurelles, organisationnelles et économiques des systèmes de santé ».

Le **Pr Éric Caumes** (Paris) a discuté du difficile sujet de la **PrEP et des IST**. Il a résumé la difficulté en une diapositive faisant la synthèse des 5 études qui se sont intéressées à ce sujet jusqu'en novembre 2023, deux montrant l'absence d'augmentation des IST, trois montrant l'augmentation des IST et les attribuant à un meilleur dépistage ou à une prise de risque accrue, sans pouvoir conclure. Il a montré, par une perspective historique, comment la prévention de l'infection VIH s’était confondue avec celles des IST dans les années 1980 et comment, à partir des années 2010, l'intérêt pour la PrEP (afin de prévenir l'infection VIH) s'est accompagné d'un désintérêt pour le « safer sex » (qui prévient toutes les IST), en dépit de la nécessité d'une prévention dite « diversifiée ». Il a rappelé les six raisons de son inquiétude: l'augmentation (peu contestable) des IST (notamment dans les populations les plus exposées), l'augmentation des complications et des séquelles des IST, la résistance croissante aux antibiotiques de bactéries courantes (gonocoque, *Mycoplasma genitalium*), l’émergence de la transmission sexuelle de maladies tropicales négligées (Monkeypox, Zika, Ébola), la réémergence des infections transmises sur le mode féco-oral (shigellose, hépatite A…), et l'indifférence vis-à-vis des violences sexuelles dont sont victimes les migrantes dans leur parcours migratoire. Il a conclu en citant le philosophe grec Thucydide (460 av. J.-C. – 400 ou 395 av. J.-C.) « l'histoire est un perpétuel recommencement » et en prônant une réhabilitation des règles du « safer sex ». Ce qui est finalement apparu logique pour achever une journée consacrée aux IST.

